# Early Exposure to Orthopaedic Surgery

**DOI:** 10.7759/cureus.57422

**Published:** 2024-04-01

**Authors:** Jhillika Patel, Divya Anand, Chandravathi Sayani, Alyanna Tam, Anna Green, Brian M Katt

**Affiliations:** 1 Department of Orthopaedic Surgery, Rutgers Robert Wood Johnson Medical School, New Brunswick, USA

**Keywords:** family connections, research, mentorship, diversity, medical education, orthopaedic surgery exposure

## Abstract

Introduction: Disparities in early orthopaedic experiences among medical students prompt a critical examination of factors influencing the availability and nature of these exposures. While the current body of literature underscores the significance of early surgical exposure and mentorship in medical education, a notable gap exists in investigating early orthopaedic exposure and its specific impact on students from diverse backgrounds.

Methods: A 16-item questionnaire, approved by our institutional review board, was administered to fourth-year medical students (MS4) and first-year orthopaedic residents (PGY-1) across U.S. orthopaedic surgery programs during the 2022-2023 application cycle. The questionnaire assessed participants' initial orthopaedic exposures and factors influencing interest in the field. Two-proportion Z-test analyses were conducted to analyze the data, and thematic analysis was used to assess qualitative data involving free-response questions.

Results: Out of 72 total respondents, the study revealed that 83% of respondents encountered orthopaedics before medical school, with initial exposures stemming from various sources such as familial connections (28%), athletics (17%), and high school or college exposures (15%), including shadowing, athletics participation, and occupation-related exposure. Disparities were observed in the availability of orthopaedic mentors and early exposure opportunities between demographic groups. Statistical analyses highlighted significant differences in access to mentors who reflected students’ identities between male and non-male participants (70% vs. 39%, p=0.02) and between white and non-white participants (69% vs. 36%, p=0.02). White participants were also more likely to first interact with a surgeon who treated them or their family members than non-white participants (35% vs 7%, p=0.04). Non-white participants were more likely than white participants to come by their first orthopaedic opportunity by searching for it independently (21% vs. 4%, p=0.03). Family and friend connections in orthopaedics were found to be influential in motivating students to pursue orthopaedics, with 40% of respondents indicating personal connections in medicine and 12% reporting family members who are orthopaedic surgeons. Research experiences were identified as important contributors to students' initial interest and motivation to ultimately pursue orthopaedics, especially those with diverse backgrounds.

Conclusion: The findings underscore the importance of early orthopaedic exposures in shaping students' interest in the field, highlighting the need for more immersive pre-clinical year opportunities and enhanced mentorship programs. Addressing disparities in mentorship access and early exposure opportunities requires systemic changes and increased support for underrepresented minorities in orthopaedics. Initiatives like mentorship programs and research opportunities can help bridge gaps in access to early orthopaedic experiences. Medical schools should prioritise targeted early access to orthopaedic exposures for all students, regardless of background. This initiative aims to promote inclusivity and cultivate a more diverse orthopaedic workforce capable of meeting the evolving healthcare needs of society.

## Introduction

There is great diversity in the scope and depth of orthopaedic experience among students pursuing orthopaedic surgery at all times in their education. This disparity prompts a critical examination of the factors influencing the availability and nature of these experiences. In recognizing the pivotal role of early orthopaedic exposure in shaping the career motivations of medical students, it is imperative to understand the current landscape, identify potential gaps, and contemplate avenues for refining educational strategies to promote early musculoskeletal experiences. Prior literature has addressed the importance of early introduction to surgery as a key driver in increasing student interest in pursuing surgical careers [1]. However, this remains to be explored for orthopaedic surgery. Existing literature underscores the significance of mentorship in medical education, revealing it as a critical determinant in shaping students' career choices [2]. However, as studies have illuminated, differences in the availability and quality of mentorship create asymmetries in opportunity, contributing to inequities in competitive specialities such as orthopaedics. Notably, a dearth of mentorship before or during medical school has been identified as a potential barrier affecting women and other minority applicants aspiring to enter surgical fields [3,4].

While many studies have concentrated on orthopaedic exposures during medical education, including clinical rotations, mentorship programs, and shadowing opportunities, there remains a prominent gap in knowledge regarding factors influencing early orthopaedic exposures [5,6]. Elements such as experiences preceding medical school, familial or friendship connections, and the timing of the first orthopaedic encounter have yet to be comprehensively explored. The need for research on these topics is particularly salient when considering its potential impact on student interest in orthopaedics, especially among underrepresented minorities. A more nuanced exploration of these multifaceted influences is essential to develop a comprehensive understanding of pathways into orthopaedics that extends beyond the medical school curriculum.

Addressing the gap in orthopaedic representation necessitates considering the various factors that influence students' early interactions with the speciality. Studies have highlighted the insufficiency of musculoskeletal education during medical school, emphasizing the importance of exposure outside formal curricular education to stimulate interest in the field [7]. Students also perceive patient-based teaching as the most valuable teaching environment, further supporting the need to evaluate and improve early clinical exposures to orthopaedic surgery [8]. Early exposure has also benefited career planning and resume building for aspiring orthopaedic surgeons [9]. Our study aims to assess the initial exposures to orthopaedic surgery of fourth-year medical students and first-year orthopaedic residents to identify factors impacting interest. Understanding these factors is crucial for developing evidence-based strategies to promote early exposure and foster inclusivity within the field.

## Materials and methods

A 16-question survey approved by our institutional review board was administered to final-year medical students (MS4) applying to orthopaedic surgery residency, and first-year residents (PGY-1) across U.S. orthopaedic surgery programs in the 2022-2023 cycle. The research team emailed orthopaedic surgery interest group leaders in U.S. allopathic medical schools, as well as program coordinators for U.S. orthopaedic surgery residency programs, with specific instructions on disseminating the survey to MS4’s applying to orthopaedic surgery for residency and PGY-1’s respectively. Medical school Orthopaedic Surgery Interest Groups (OSIG) serve to support and stimulate students' interest in pursuing orthopaedics and exist across the majority of medical schools in the United States. Student leaders are chosen each year to lead this group at each respective school. Using OSIG student leader contact information listed on each school's website, the research team sent the survey to the student leaders with instructions to share with M4s who applied into orthopaedic surgery. No additional incentives were provided to participants to complete the survey.

Two-proportion Z-test analyses were performed to determine differences between the responses of participants grouped by self-identified gender (male vs. non-male) and race (white vs. non-white). Thematic analysis was used to analyze free-response questions.

## Results

Seventy-two people in total responded to the survey, of whom 23 were female, 47 were male, and two were genderqueer. Fifty-three people were white, three were black or African-American, 11 were Asian, one was Native American or Alaska Native, and four self-identified as "other." Eight self-identified their ethnicity as Hispanic or Latino (Table 1).

**Table 1:** Participant demographics

Variable	n	%
Gender		
Male	47	65%
Non-male	25	35%
Race		
White	53	74%
Non-White	19	26%
Gender and Race		
Male and White	35	49%
Male and Non-White	12	17%
Non-male and White	18	25%
Non-male and Non-White	7	10%

Initial exposures and motivational experiences to pursue orthopaedics 

The study’s primary outcome sought to assess the timing and impact of the respondents’ first orthopaedic experience on their decision to pursue the field. Initial exposures refer to experiences or encounters that notably enhance participants' knowledge and comprehension of orthopaedics as a medical speciality. Among 72 respondents, 83% encountered orthopaedics before medical school, either during high school or earlier (28%), undergraduate studies (43%), gap years (8%), or graduate school (5%) (Table 2). Initial exposures to the field stemmed most frequently from family and friend connections (28%), followed by athletics (17%), medical school resources (17%), and high school and college experiences (15%). Other means of initial exposure included clinical rotations (3%), independent searching for surgeons to shadow (8%), experience as an orthopaedic patient (6%), and orthopaedics-related employment (6%) (Table 3).

**Table 2:** When was your first encounter with orthopaedics?

Variable	Total responses	Male	Non-male	p-value	White	Non-White	p-value
High School	28%	23%	39%	0.20	33%	7%	0.05
Undergraduate School	43%	43%	44%	0.94	41%	50%	0.55
Graduate School	5%	6%	0%	0.29	6%	0%	0.35
Gap Year	8%	9%	6%	0.69	6%	14%	0.32
Medical School	17%	19%	11%	0.44	14%	29%	0.19

**Table 3:** How did you get your first orthopaedic encounter opportunity? * = significant finding

Variable	Total Responses	Male	Non-male	p-value	White		Non-White	p-value
Family/Friends	28%	22%	44%	0.06		25%	36%	0.41
Athletics	17%	17%	17%	1		22%	0%	0.05
High School/College	15%	17%	11%	0.55		16%	14%	0.86
Medical School Resources	17%	17%	17%	1		16%	21%	0.66
Clinical Rotations	3%	4%	0%	0.40		4%	0%	0.45
Independent Searching	8%	11%	0%	0.14		4%	21%	0.03*
Employment	6%	6%	6%	1		6%	7%	0.87
Other (Patient Experiences)	6%	6%	6%	1		8%	0%	0.28

Subgroup analysis between males and non-males revealed no significant differences within each selected initial orthopaedics experience. However, there was a notably greater proportion of white participants (33%) than non-white participants (7%) reporting initial exposure to orthopaedics in high school or earlier (p=0.05) (Table 2). A significantly greater proportion of non-white (21%) than white (4%) students obtained their first orthopaedic encounter by independently searching for it, such as by contacting surgeons to ask to shadow (p=0.03) (Table 3).

Additionally, 44% of non-male participants cited their first orthopaedics encounter through family and friend connections, while 22% of male participants cited the same (p=0.06) (Table 3). While not a statistically significant finding, this may suggest that non-male students rely more on personal connections for their earliest field exposures.

Participants wrote free responses when asked what their first encounter with orthopaedics entailed, and thematic analysis was performed. Shadowing and intentional career exploration (44%) was the most common first encounter, followed by experiences in which the respondent or a family member was an orthopaedic patient (27%) (Table 4). Other common responses included employment and internship experiences, research projects, and encounters with friends and family in the field. Subgroup analysis showed that 32% of responses from white participants cited patient experiences as their initial orthopaedic encounters, while only 7% of non-white respondents cited the same. More non-white than white respondents cited research experiences as their initial encounter with the field (p=0.02) (Table 4).

**Table 4:** What did your first encounter with orthopaedics entail? * = significant finding

Variable	Total Responses	Male (n=47)	Non-male (n=18)	p-value	White (n=51)	Non-White (n=14)	p-value
Personal Connections	12%	13%	9%	0.62	12%	13%	0.91
Patient/Family Member Orthopaedic Patient Experiences	27%	25%	30%	0.65	32%	7%	0.05
Employment/Internships	11%	10%	13%	0.70	8%	20%	0.17
Shadowing/Career	44%	44%	43%	0.94	45%	40%	0.73
Research	7%	8%	4%	0.52	3%	20%	0.02*

While 38% percent of the respondents reported that the first orthopaedic healthcare provider they interacted with was a surgeon they individually contacted, 29% reported first interacting with an orthopaedic surgeon who treated them or their family member (Table 5). Significantly more white participants (35%) than non-white participants (7%) interacted first with an orthopaedic surgeon they or their family was treated by (Table 5).

**Table 5:** Who was the first orthopaedics health professional you interacted with, either in person or virtually? * = significant finding

Variable	Total Responses	Male	Non-Male	p-value	White	Non-White	p-value
Surgeon/Physician you individually contacted	38%	43%	28%	0.27	35%	50%	0.31
A surgeon that you/family/friend was treated by	29%	23%	44%	0.09	35%	7%	0.04*
Family or friend in the orthopaedics field	17%	21%	6%	0.15	18%	14%	0.73
Other	15%	13%	22%	0.37	12%	29%	0.12

Among all survey participants, 48% felt that an athletic history or current athlete status was integral to early exposure to the field (Table 6). Furthermore, 29% of all participants attributed personal injury management as contributing to their interest in orthopaedics (Table 7). Subgroup analysis revealed that all participants who got their first exposure to orthopaedics through athletics were white (p=0.05) (Table 3). While not statistically significant, this may suggest that participation in athletics and subsequent exploration of orthopaedics is more available to white populations, creating more disparity in access points to orthopaedic surgery experiences.

**Table 6:** Was an athletic history or current athlete status part of your initial exposure to orthopaedics?

Variable	Total Responses	Male	Non-male	p-value	White	Non-White	p-value
Yes	48%	45%	56%	0.43	51%	36%	0.31
No	52%	55%	44%	0.43	49%	64%	0.31

**Table 7:** Was an injury integral in your exposure to orthopaedics?

Variable	Total Responses	Male	Non-male	p-value	White	Non-White	p-value
Yes	29%	30%	28%	0.87	31%	21%	0.47
No	71%	70%	72%	0.87	69%	79%	0.85

Thirty-four percent of participants stated that most of their orthopaedic experience prior to clinical rotations was from shadowing, while 22% stated research and 11% stated interest groups. Fifty-percent people who stated that research was the majority of their experience were non-white, and 14% were white (p=0.004) (Table 8).

**Table 8:** What was the majority of your orthopaedic surgery experiences before medical school rotations? * = significant finding

Variable	Total Responses	Male	Non-male	p-value	White	Non-White	p-value
Pre-medicine (during Undergraduate Studies)	8%	9%	6%	0.69	8%	7%	0.90
Pre-clerkship Years	9%	9%	11%	0.80	12%	0%	0.17
Orthopaedic Interest Groups	11%	9%	17%	0.36	14%	0%	0.14
Shadowing	34%	32%	39%	0.60	37%	21%	0.26
Research	22%	23%	17%	0.60	14%	50%	0.004*
Conferences	2%	2%	0%	0.55	2%	0%	0.60
Summer program	2%	2%	0%	0.55	0%	7%	0.06
Employment	8%	6%	11%	0.49	8%	7%	0.93
Injury	2%	2%	0%	0.55	2%	0%	0.61
Little to no experience	5%	6%	0%	0.29	4%	7%	0.61

The majority of participants seriously considered pursuing orthopaedics in their M1 year (first year of medical school) or earlier (69%), while 20% decided in their M3 year (third year of medical school), and 11% decided in their M2 year (second year of medical school) (Table 9). Most considered pursuing orthopaedics well before their focused clinical years of medical school. However, the strongest influencing factor for students' final decision to pursue orthopaedics stemmed from rotations (37%), where students focus on specialities such as surgery or take orthopaedic electives for a focused period of time (Table 10). Mentorship, shadowing, family and friends in orthopaedics, and electives/away rotations were other reported significant influences on participants' decision to pursue orthopaedic surgery.

**Table 9:** When in your medical school journey did you seriously start considering orthopaedics?

Variable	Total Responses	Male (n=47)	Non-male (n=18)	p-value	White (n=51)	Non-White (n=14)	p-value
M1	69%	72%	61%	0.38	67%	79%	0.40
M2	11%	11%	11%	0.95	10%	14%	0.63
M3	20%	17%	28%	0.33	24%	7%	0.16

**Table 10:** Which type of experience motivated you the most in choosing to pursue orthopaedics?

Variable	Total Responses	Male	Non-male	p-value	White	Non-White	p-value
Family/Friends in orthopaedics	9%	11%	6%	0.53	8%	14%	0.46
Mentorship	25%	19%	39%	0.09	24%	29%	0.70
Clinical Rotations	37%	34%	44%	0.44	39%	29%	0.47
Shadowing	20%	23%	11%	0.27	18%	29%	0.36
Electives/Away Rotations	8%	11%	0%	0.15	10%	0%	0.22
Research	2%	2%	0%	0.54	2%	0%	0.60

Family and friend influences and mentorship in orthopaedics

When investigating the influences of family and friend connections in orthopaedics, 40% of respondents indicated personal connections in medicine, and 12% of applicants reported having family members who are orthopaedic surgeons (Table 11). Though most respondents listed traditional experiences (37% rotations, 25% mentorship, and 20% shadowing) as their primary motivators to pursue orthopaedics, 9% cited family and friends in the field as their primary motivators (Table 10).

**Table 11:** Do you have any family members/friends in orthopaedics or another medical speciality? Were they integral to your exposure? * = significant finding

Variable	Total Responses	Male	Non-male	p-value	White	Non-White	p-value
Yes							
Orthopaedics	12%	15%	6%	0.33	16%	0%	0.11
Integral to exposure	75%						
Another specialty	28%	17%	56%	0.002*	24%	43%	0.16
Integral to exposure	44%						
No	60%	68%	39%	0.03*	61%	57%	0.79

Of the eight respondents with prior connections to orthopaedics, seven were male, and all eight were white. 75% of respondents with orthopaedic connections and 44% of those with non-orthopaedic connections in medicine found these connections to be integral to their exposure to the field. Notably, a significantly greater proportion of non-male respondents had connections in non-orthopaedic medical specialities than male participants (p=0.002), and a greater proportion of male respondents (68%) had no medical field connection at all (p=0.03) (Table 11).

In evaluating mentorship, 38% of participants reported a significant absence of mentors who reflected their identity. Mentorship emerged as a commonly cited subjective barrier to early exposure in orthopaedics (Table 12). Among participants, 70% of males claimed access to mentors reflecting their personal identity, whereas only 39% of non-male participants reported such access (p=0.02) (Table 12). Similarly, 69% of white participants had access to identity-affirming mentors, compared to 36% of non-white participants (p=0.02) (Table 12). In response to inquiries about the primary influencing factor in their decision to pursue orthopaedic surgery residency, 25% of students pinpointed mentorship as the most influential aspect of their experience (Table 10).

**Table 12:** Did you have access to a mentor who reflects your personal identity (race, gender, etc.)? * = significant finding

Variable	Total Responses	Male	Non-male	p-value	White	Non-White	p-value
Yes	62%	70%	39%	0.02*	69%	36%	0.02*
No	38%	30%	61%	0.02*	31%	64%	0.02*

Barriers to orthopaedics exposure 

When asked about any encountered barriers to orthopaedic surgery exposure, most respondents reported no barriers (55%). The most common barrier to exposure cited was personal and social factors (15%), including a lack of personal connections in the field, first-generation college student status, and subjective feelings of lack of support. Thirteen percent of respondents listed a need for more organized resources, including an orthopaedic residency program affiliated with their medical school and insufficient resources to support the number of students interested in orthopaedics. Lack of mentorship, shadowing, and research opportunities were also commonly described (Table 13).

**Table 13:** Did you have any barriers to orthopaedic surgery exposure? If so, what was the barrier? * = significant finding

Variable	Total responses	Male	Non-male	p-value	White	Non-White	p-value
Yes							
Barriers to early exposure	10%	23%	0%	0.11	13%	0%	0.13
Lack of organized resources	13%	20%	17%	0.56	11%	19%	0.41
Lack of mentors and shadowing opportunities	7%	13%	6%	0.77	7%	6%	0.89
Limited research opportunities	4%	10%	0%	0.30	5%	0%	0.34
Personal and social factors	15%	33%	6%	0.19	10%	31%	0.048*
No	55%	0%	72%	0.03*	53%	44%	0.58

## Discussion

The responses in this study suggest that early exposure prior to medical school is exceedingly common and integral to the decision to pursue orthopaedics. Very early exposure to orthopaedics may be disproportionately available to white populations than non-white populations in the form of personal connections and patient personal experiences. Studies show that orthopaedic surgeries, including total knee arthroplasty, total shoulder arthroplasty, and surgical clavicular fracture repair, occur at higher rates in white populations than minority populations, including Black, Hispanic, Asian, Native American, and mixed-race populations [10,11]. Patient experiences accounted for a significant proportion of respondents’ initial exposures to the field, highlighting an early opportunity to learn about careers in orthopaedics that minority students likely do not have equal access to. From responses to questions about the timing of early exposure, exposures prior to medical school led participants to consider pursuing orthopaedics by their first year. Rather than making a choice in the third year, the traditional time to make a decision on which medical speciality to pursue, students applying to orthopaedics have two additional years to build their applications for the field [12].

Many participants’ very early exposures predominantly stemmed from opportunities presented to them from their circumstances or activities in life. Respondents who were first exposed to orthopaedic surgery during high school or earlier drew on a variety of experiences, including involvement in athletics, personal connections, patient encounters, employment, and their high school environment. While many initial experiences were shadowing, only 8% were found by independent searching. However, that 8% is made up primarily of non-white students. This highlights a difference in both when and how white vs. minority students gain exposure to orthopaedics; white students have earlier access to orthopaedics upon which to build further experiences and enhance interest, whereas non-white students actively seek opportunities later in their education. Orthopaedic surgery shadowing and educational programs in high schools in diverse school districts can help bridge the gap in access to early orthopaedic experiences.

While most medical students do not formally pursue athletics during medical school training, the reported majority of students' athletic experience is likely from before medical school. This issue prompts an investigation into whether medical students with prior involvement in athletics gravitate towards orthopaedics due to its connection with sports. Alternatively, early experiences with sports-related injuries and treatment from orthopaedic surgeons may foster an early awareness of orthopaedic surgery as a career path. This motivates young athletes to pursue medical training with the aspiration of becoming orthopaedic surgeons.

Orthopaedic connections played a critical role in early exposure to the field for those to whom those connections were available. However, the findings suggest that existing connections to the medicine of any speciality increase awareness of careers in medicine, including orthopaedics, facilitate exposure to orthopaedics, and encourage non-male medical students to pursue the field. It also suggests that minorities may rely on these connections for exposure more than non-minorities. 

Students who decide to pursue orthopaedic surgery during their third year may encounter additional barriers due to less time to develop a reserve of orthopaedics knowledge, connect with mentors, shadow surgeons, and conduct research in orthopaedics. This may lead to a weaker curriculum vitae prior to the time of orthopaedic surgery residency applications. On the other hand, students who have decided to pursue the field early on have a head start in acquiring these clinical and research experiences, which can make them stronger residency candidates. While developing interest in the field during the third year of medical school can still lead to a successful orthopaedic residency match and clinical skill development, it may leave students without previous experience lagging in knowledge critical to being an effective sub-internship student or lead to disadvantages with the process of building their orthopaedics residency application. 

For many years, research in orthopaedics has served as a way to demonstrate interest, contribute to the field, build professional connections, gain knowledge, and strengthen residency applications. Students belonging to racial and ethnic minorities who could not accrue earlier meaningful experiences in orthopaedics can use research as a means of career involvement in tandem with medical school activities. Organized research programs in medical school that are easily accessible and noncompetitive can help interested students gain experience and connections before starting clinical rotations.

It is established in the existing literature that among minority students, social factors can greatly influence one’s decision to apply to orthopaedics, specifically through mentorship and the availability of mentors that match applicants’ gender and race [13]. Increasing the availability of non-male and racial minority mentors in orthopaedic surgery fosters a welcoming environment for non-male and minority students. It may also improve the quality of mentorship by allowing specific advice about shared social and environmental experiences in the orthopaedic application process [4]. Further, ethnic representation has been cited in the literature to increase feelings of belongingness, inspiration, individual self-efficacy, and subjective feelings of support [14-16]. Increasing the presence of underrepresented minority faculty and residents has been associated with a higher likelihood of underrepresented minority medical students applying to orthopaedics [17]. Multiple participants cited a lack of family members in medicine, a lack of structured mentorship programs, and difficulty shadowing during their first year of medical school as barriers to orthopaedics exposure. As personal and social barriers to residency application disproportionately affect minority students, we must focus on dissolving them on a systemic level.

Limitations to this research include a low percentage rate of responses, which limits the generalizability of results. In addition, students who elected not to pursue orthopaedic surgery may have experienced barriers or lack of exposure and have been dissuaded from pursuing the field. These individuals did not participate in the study and, therefore, are not represented in the presented results. Future studies can look into factors that dissuade students from applying to orthopaedic surgery. This can be done by following incoming first-year medical students into their fourth year, comparing their initial speciality interest with their final decision, and asking them what factors persuaded them for or against applying to orthopaedics. Other studies can implement structured research or mentorship programs early on in medical school and observe how it impacts the number of applicants into orthopaedics compared to previous years. These further studies are crucial in building onto the growing body of evidence of the importance of the factors mentioned above, which will result in more motivation to make changes to help aspiring orthopaedic surgeons have the resources to make an informed decision.

## Conclusions

These findings underscore the significance of early orthopaedic exposure, with most students’ first encounters before medical school. However, not all aspiring applicants have access to such opportunities to explore the field, leading to differences in representation and emphasizing the need for proactive measures. Opportunities for orthopaedic patient experiences, athletic participation, and personal connections to orthopaedic surgeons that are disproportionately available to white students must be counterbalanced by organized efforts to introduce young students from diverse backgrounds to the field. Orthopaedic organizations that support underrepresented minorities, such as the Perry Initiative, Ruth Jackson Orthopaedic Society, Nth Dimensions, and J Robert Gladden Orthopaedic Society, are actively increasing access to orthopaedic experiences through mentorship and networking. Medical schools should also adopt similar programs to provide earlier access to clinical orthopaedics, mentorship, networking, and research opportunities, especially in the preclinical years. Acknowledging the later and limited role medical schools play in students’ initial exposures to orthopaedics, collaborating with various high schools and colleges through career exploration and professional development programs can further broaden the accessibility of orthopaedic experiences for students from all backgrounds. By understanding the multifaceted influences on students' perceptions and ultimate decision-making to pursue orthopaedics, medical educators can develop targeted interventions to cultivate a more diverse and skilled orthopaedic workforce.

## Appendices

Survey questions

Demographics

**Age**: slide to select

**Gender**:

o Cis female  

o Cis male  

o Trans female  

o Trans male  

o Non-binary  

o Genderqueer/gender-nonconforming  

o Other (please specify)   __________________________________________________

Sex assigned at birth:

o Male  

o Female 

o Other (please specify)  __________________________________________________

Sexual orientation:

o Heterosexual  

o Gay  

o Lesbian  

o Bisexual  

o Pansexual  

o Asexual  

o Other (please specify)  __________________________________________________

Race/Ethnicity:

o White  

o Black or African American  

o Asian  

o Native American or Alaska Native  

o Native Hawaiian or Pacific Islander  

o Other  

Are you of Hispanic or Latino origin?

o Hispanic or Latino  

o Not Hispanic or Latino  

Exposure questionnaire 

(1/16) When was your first encounter with orthopaedics?

o High school or earlier  

o Undergraduate studies  

o Graduate school  

o Gap years before medical school  

o During medical school  

(2/16) How did you get your first orthopaedic encounter opportunity?

o Through family or friend connections  

o Through resources at medical school  

o Through clinical rotations  

o Through external organizations (AMA, AAOS, RJOS etc.)  

o Through high school or college  

o Independent searching  

o Through athletics 

o Through employment  

o Other  __________________________________________________

 **(3/16) What did your first encounter with orthopaedics entail? (few words)**

________________________________________________________________

(4/16) Who was the first orthopaedics health care professional you interacted with, either in person or virtually?

o Family or friend in orthopaedic surgery  

o A surgeon/physician you individually contacted  

o A surgeon/physician you or a family member/friend was treated by  

o Other (please describe)  __________________________________________________

(5/16) Do you have any family members/friends in orthopaedics or another medical specialty?

o Orthopaedics  

o Other specialty   __________________________________________________

o N/A  

(6/16) If so, were these family/friends integral to your exposure to orthopaedics?

o Yes  

o No  

o N/A  

(7/16) Was an athletic history or current athlete status part of your initial exposure to orthopaedics?

o Yes  

o No  

(8/16) Was an injury integral in your exposure to orthopaedics?

o Yes  

o No  

(9/16) Did you have access to a mentor who reflects your personal identity (gender, race, etc)?

o Yes  

o No  

(10/16) What has the majority of your orthopaedic surgery experience thus far been from?

o Residency  

o Required medical school rotations  

o Electives/Away Rotations  

o Interest group involvement  

o Pre-clerkship curriculum/anatomy lab  

o Conferences and workshops  

o Research involvement  

o Shadowing set up independently  

o Other (please describe)   __________________________________________________

(11/16) What was the majority of your orthopaedic surgery experience BEFORE medical school rotations from?

o Interest group involvement  

o Pre-clerkship curriculum/anatomy lab  

o Pre-med years in undergrad  

o Conferences and workshops  

o Research involvement  

o Shadowing set up independently  

o Summer programs  

o Other (please describe)  __________________________________________________

(12/16) When in your medical school journey did you seriously start considering orthopaedic surgery?

o M1  

o M2  

o M3  

o M4  

(13/16) Did you have any barriers to orthopaedic surgery exposure? If so, describe:

________________________________________________________________

(14/16) If you had to pick one aspect from your orthopaedic experiences that motivated you the most to pursue orthopaedics, what would it be?

o Family / Friend in orthopaedics  

o Mentorship  

o Research  

o Shadowing  

o Rotations  

o Electives/Away Rotations  

o Conferences and Workshops  

o Interest group involvement  

(15/16) Are any of your close relatives/family members orthopaedic surgeons? If yes, state how many. 

o Yes (please share how many)   __________________________________________________

o No  

(16/16) Are any of your close relatives/family involved in orthopaedics in another capacity (for ex. Resident, Physician Assistant, Industry, Research, Athletic Teams, other)? If yes, please describe in a few words. If no, N/A.

________________________________________________________________
